# Reduced penetrance in familial Avellino corneal dystrophy associated with *TGFBI* mutations

**Published:** 2009-01-14

**Authors:** Wenping Cao, Hongyan Ge, Xiaobo Cui, Lu Zhang, Jing Bai, Songbin Fu, Ping Liu

**Affiliations:** 1 Eye hospital, The First Affiliated Hospital, Harbin Medical University, Harbin, China; 2 Laboratory of Medical Genetics, Harbin Medical University, Harbin, China

## Abstract

**Purpose:**

To characterize the clinical phenotype, histopathological features, and molecular genetic basis of an Avellino corneal dystrophy (ACD) in a Chinese family.

**Methods:**

A complete ophthalmologic examination was performed in 21 individuals (6 affected and 15 unaffected) of the four-generation family. DNA was obtained from peripheral blood leukocytes of each participant. Genetic analysis included *TGFBI* polymerase chain reaction (PCR) amplification and automated nucleotidic sequenceing of all 17 exons of genomic DNA. Histological analysis of corneal tissue from the proband was performed after a penetrating keratoplasty. One hundred Chinese controls were scanned for the presence of the R124H mutation by amplifying *TGFBI* exon 4 and then by direct sequencing of PCR products.

**Results:**

The proband of the pedigree had phenotypic features consistent with diagnosis of ACD. He was homozygous for the same R124H mutation in *TGFBI* as previously reported in Japan and European countries. In addition, 4 affected and 7 unaffected individuals carried the same variation in the heterozygous state were identified. None of the 100 control subjects was positive for this mutation. Moreover, a variable expressivity and an apparent non-penetrance were observed in the individuals with heterozygous R124H mutation in our pedigree. After excluding the missed diagnosis or a late onset, it could be interpreted as a reduced penetrance.

**Conclusions:**

We reported a novel ACD family which exhibited a reduced penetrance of phenotype in northern China. This outcome supports that although the R124H mutation is one of the genetic causes of the disease, different genetic and environmental factors may influence the expressivity and the penetrance. Uncovering the mechanism may facilitate us to inhibit the occurrence of the corneal dystrophy caused by the R124H mutation in *TGFBI*, irrespective of the homozygous and heterozygous mutation.

## Introduction

Corneal dystrophies (CDs) are hereditary diseases involving the formation of corneal opacities on different layers of the cornea, which lead to significant impairment of corneal transparency and refraction [1]. In 1997, Munier et al. [2] identified the gene on chromosome 5q31 responsible for these autosomal dominant corneal dystrophies: transforming growth factor beta-induced (*TGFBI*, OMIM 601692, formerly called *BIGH3*). Four separate mutations were discovered, resulting in four distinct corneal dystrophies including granular dystrophy (GCD; R555W), lattice dystrophy type I (LCD-I; R124C), Avellino dystrophy (ACD; R124H) and Reis-Bücklers dystrophy (RBCD; R555Q).

ACD (also known as GCD-II) is one of the *TGFBI* associated corneal dystrophies, of which the clinical aspect is the coexistence of granular deposits and histological amyloidal deposits in the cornea [3]. The name of the dystrophy comes from Avellino, Italy – a city where three families affected with the disease originated [4]. It is characterized clinically by corneal opacities that are shaped like rings, disks, stars, and snowflakes. Early clinical symptoms of ACD appear during the first or second decade of life. Linear opacities may be present [5,6], but the typical lines of LCD are usually absent. Compared to GCD-I, the progression of ACD is delayed and slower and the visual acuity is less impaired. In the absence of a histopathologic evaluation or a examination of the molecular genetic defect, ACD can be difficult to be distinguished from GCD-I. In Japanese individuals, ACD is the most common hereditary corneal dystrophy, responsible for 72% of corneal dystrophies associated with *TGFBI* [7]. The cause of this unique spectrum is not clear.

To date, all ACD cases studied with molecular genetic technique have demonstrated a mutation in codon 124 of *TGFBI* in which histidine replaces arginine (R124H) [8-11]. The disease is more severe in homozygous patients than in their heterozygous counterparts [10,11]. In this study, we report a novel family of ACD with the same mutation that exhibited a reduced penetrance of phenotype in northern China.

## Methods

### Patients

**Figure 1:**
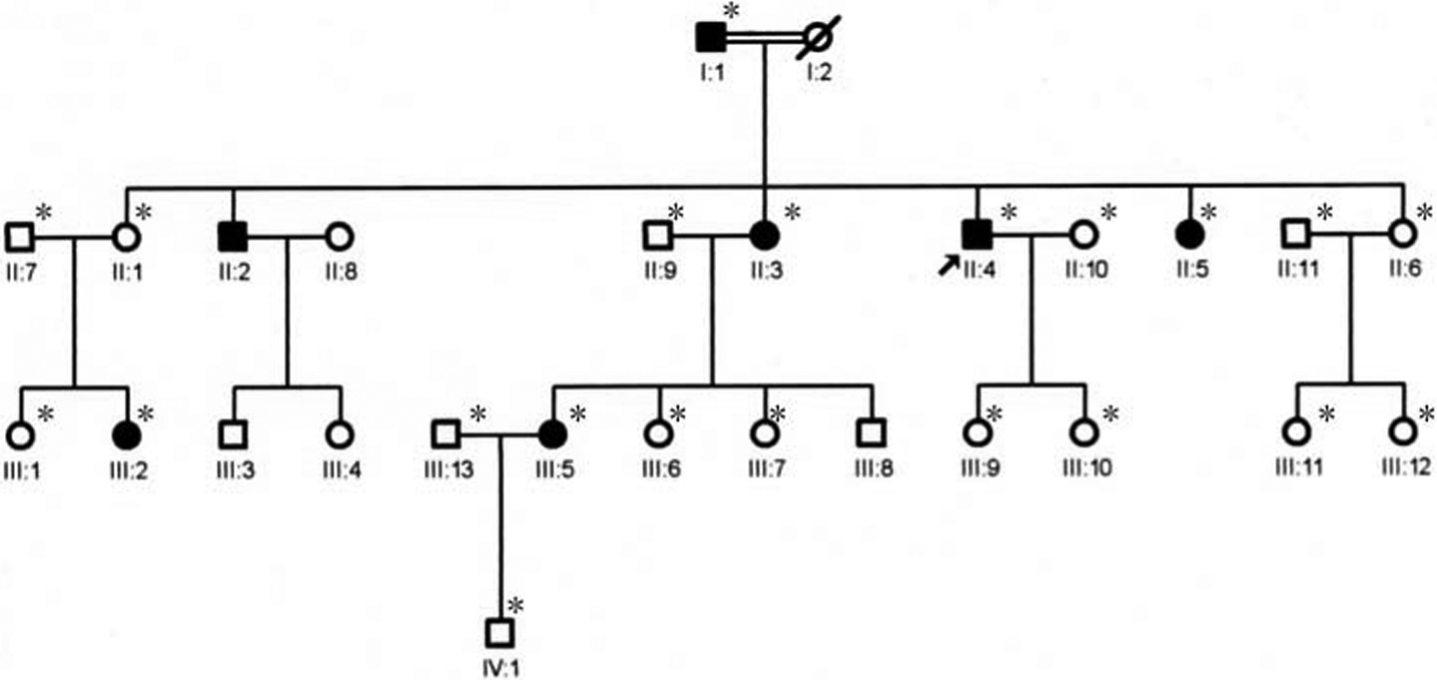
Pedigree showing a four-generation family affected by ACD. Circles represent females, squares represent males. The arrow indicates the proband, and the filled symbols indicate affected individuals. Asterisks indicate members of the family who underwent clinical examination and molecular analyses.

A four-generation ACD family was collected from the Heilongjiang province in northern China (Figure 1). Informed consent which adhered to the Declaration of Helsinki and the Heilongjiang Institutional Review Board was obtained from all participants. Twenty-one members of the family were enrolled, 6 affected and 15 unaffected. The ages ranged from 2 to 74 years. All the members (except IV1) were examined over 3 years. One hundred unrelated healthy volunteers were recruited as controls.

### Clinical evaluation

The affected status of all participating members was determined by extensive ophthalmologic examinations, which included best corrected visual acuity test, slit-lamp examination, corneal thickness measurement, intraocular pressure measurement, and dilated fundus examination. Detailed clinical histories were obtained and particular attention was paid to the age of onset, initial signs and symptoms, progression of disease, and other ocular therapeutic procedures. The corneal phenotypes of affected individuals were documented by slit-lamp photography and assessed by at least two investigators ignorant of the genotypes.

### Molecular analysis

Peripheral blood was collected from each of the 6 affected and 15 unaffected family members, and genomic DNA was extracted using a QIAamp DNA Blood Mini Kit (Qiagen, Hilden, Germany). All 17 exons of *TGFBI* were analyzed by the polymerase chain reaction (PCR) amplification and DNA sequencing. The forward and reverse primers and the PCR conditions we used were as described by Afshari et al. [12] and Munier et al. [2,13]. The PCR products were purified with a QIAquick PCR Purification Kit (Qiagen, Valencia, CA) and sequenced on both strands using the ABI BigDye Terminator Cycle Sequencing kit v3.1, (ABI Applied Biosystems, Foster City, CA). Sequence results were compared with the wild type *TGFBI* sequence (GenBank AY149344). The one hundred Chinese controls were scanned for the presence of the R124H mutation by amplifying the exon 4 and then by direct sequencing of PCR products.

### Histologic evaluation

Corneal tissue obtained at penetrating keratoplasty was available from the proband for histologic study. Tissue sections were examined by light microscopy after being stained with hematoxylin-eosin (H&E), Masson’s trichrome, periodic acid Schiff (PAS), and Congo red.

## Results

### Clinical findings

Clinical features of family members are summarized in Table 1. The age at onset for affected individuals was between 9 and 28, with a mean of 18.7–19.3 years. At onset, symptoms related to either visual loss or foreign body sensation, both of which were usually bilateral at that stage. The progress of corneal opacities in affected members was commonly symmetric.

**Table 1:** Clinical features in family members with the *TGFB1 *R124H mutation.

**Individual case**	**Gender**	**Age**	**Status**	***TGFBI* genotype**	**Age at onset**	**Symptoms at onset**	**Visual acuity at presentation**	
							**OD**	**OS**
I1	M	74	Affected	wt/R124H	24–28	FBS	1.0	1.0
II1	F	56	Unaffected	wt/R124H	-	-	1.0	1.2
II3	F	49	Affected	R124H/ R124H	9	VA↓FBS. Ph	10cm/CF	10cm/CF
II4	M	43	Affected	R124H/ R124H	11	VA↓FBS. Ph	20cm/CF	20cm/CF
II5	F	41	Affected	wt/R124H	21	VA↓FBS	0.8	0.8
II6	F	37	Unaffected	wt/R124H	-	-	1.0	1.0
III2	F	29	Affected	wt/R124H	22	FBS	1.0	1.0
III5	F	27	Affected	wt/R124H	25	FBS	1.0	1.0
III6	F	23	Unaffected	wt/R124H	-	-	1.0	1.0
III7	F	21	Unaffected	wt/R124H	-	-	0.8	1.0
III9	F	13	Unaffected	wt/R124H	-	-	1.0	1.0
III10	F	4	Unaffected	wt/R124H	-	-	1.0	1.0
III11	F	11	Unaffected	wt/R124H	-	-	1.0	1.0

M: male, F: female, OD: right eye, OS: left eye, wt: wild type, wt/R124H: heterozygous R124H mutation, R124H/R124H: homozygous R124H mutation, VA↓: visual acuity decreased, FBS: foreign body sensation, Ph: photophobia, CF: count finger. Information that is not provided about clinically unaffected individuals is marked with a dash (-).

**Figure 2:**
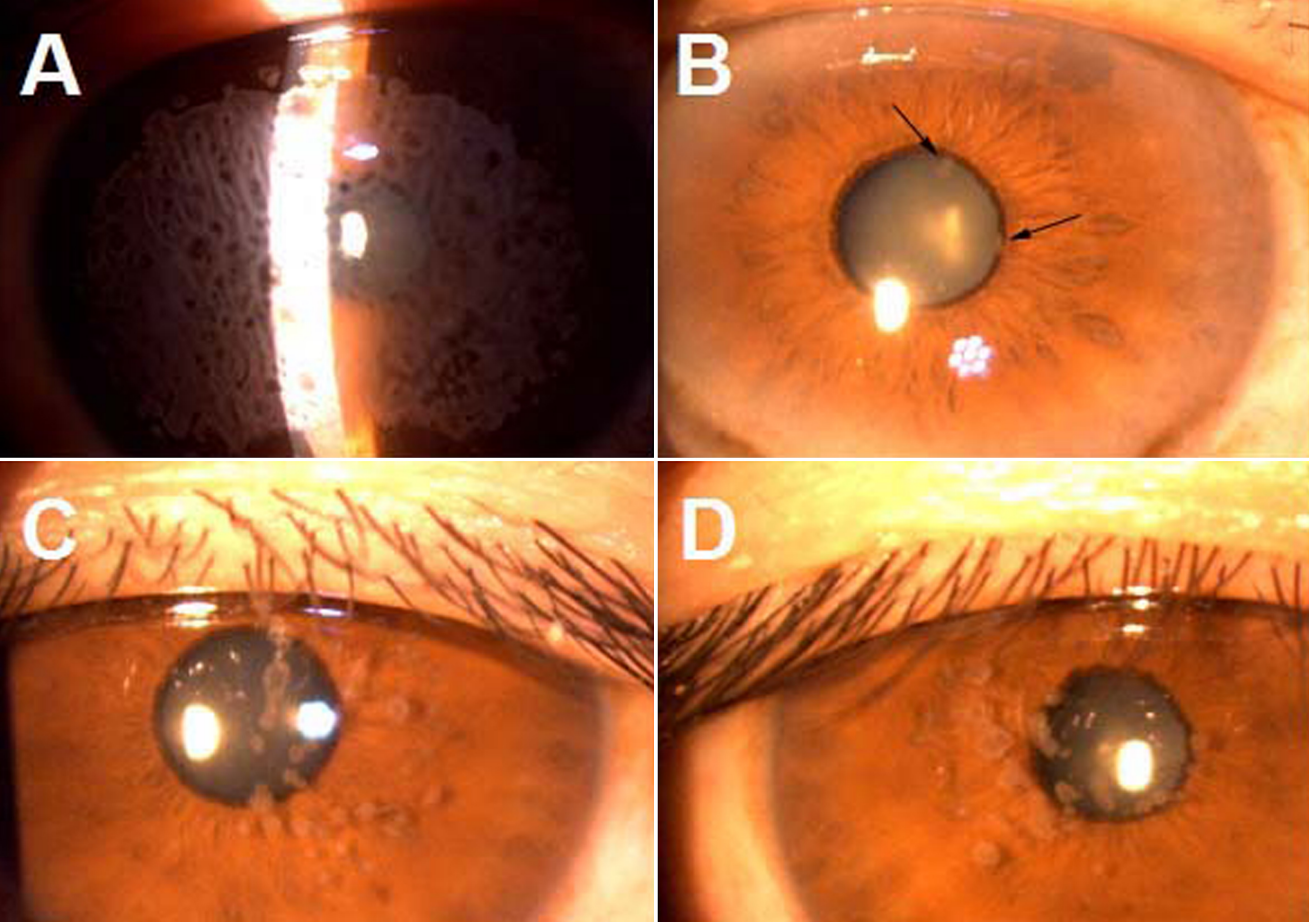
Corneal phenotype of the affected family members with ACD due to an R124H mutation in *TGFBI*. **A**: Preoperative photograph of the right eye of proband revealed grayish spot-like confluent opacities presented in the anterior stroma and covering almost the entire cornea. **B**: Corneal examination of the left eye of I1 (father of the proband) showed several distinct granular deposits in the superficial stroma of the central cornea (arrows). **C** and **D**: Multiple granular deposits and a few confluent opacities were observed in the anterior stroma of central cornea in both right eyes of III2 and III5, respectively.

The proband (II4) was a 43-year-old man who was the offspring of a consanguineous marriage. He was 11 years old when he had bilateral foreign body sensation and a visual defect. He had experienced progressive deterioration in visual acuity bilateral over the preceding 32 years. Corneal examination revealed grayish spot-like confluent opacities presented in the anterior stroma and covered almost the entire cornea (Figure 2A). Ultimately, penetrating keratoplasty was performed on his right eye at the age of 43. Visual acuity was 20 cm/CF in both eyes before the operation.

The father of the proband (I1) had his initial symptom at about 24–28 years old and began with a mild foreign body sensation. His visual acuity was 1.0 in both eyes when he was 74 years old. Slit-lamp examination showed several distinct granular deposits in superficial stroma of the central cornea (Figure 2B).

The earliest symptoms for III2 and III5 was foreign body sensation as well. The ages of onset were 22 and 25, respectively. Multiple granular deposits and a few confluent opacities were observed in the anterior stroma of central cornea (Figure 2C,D). No visual defect was complained about.

### Molecular genetic analysis

**Figure 3:**
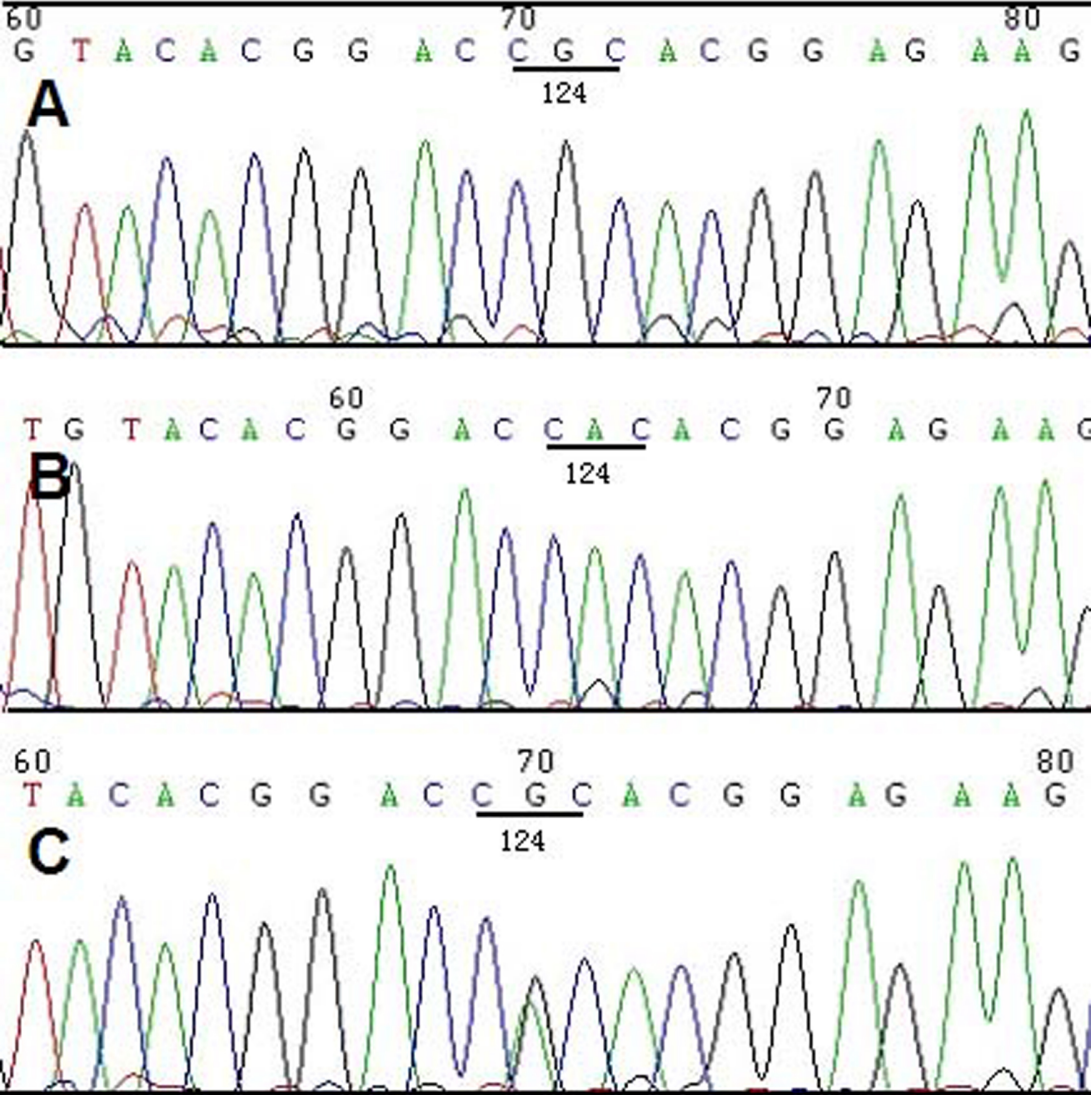
Sequence analysis of *TGFBI* exon 4 in this family. **A**: A normal sequence is shown, which encodes arginine in codon 124. **B**: The proband had homozygous substitutions at nucleotide position c.371G>A, which results in a arginine to histidine acid substitution (R124H). **C**: The proband’s father had a heterozygous mutation of R124H at the same position.

After direct sequencing of *TGFBI* exons, a homozygous sequence variation was detected at nucleotide position c.371G>A in II3 and II4 (Figure 3B).The variation lies in exon 4 and results in a arginine to histidine acid substitution at the protein level (R124H). In addition, 4 affected (I1, II5, III2, and III5) and 7 unaffected (II1, II6, III6, III7, III9, III10, and III11)family members carrying the same variation in the heterozygous state were identified(Figure 3C) . None of the 100 control subjects was positive for this mutation.

### Histologic evaluation

**Figure 4:**
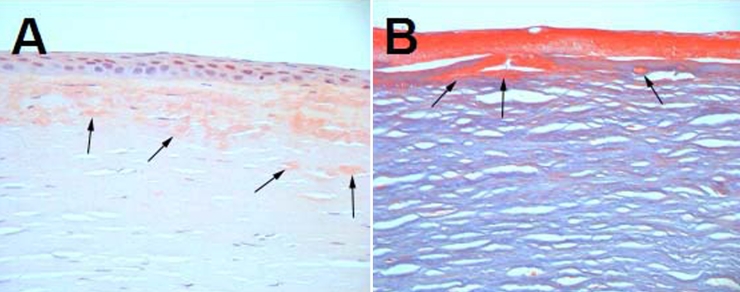
Histopathological features of affected cornea from the right eye of the proband carrying the homozygous *TGFBI* R124H mutation at the age of 43. The visual acuity of the proband before penetrating keratoplasty was 20 cm/CF. **A**: Light microscopy of excised corneal tissue stained with congo red revealed variably sized and irregularly shaped deposits of amyloid material, predominantly in the anterior and middle stroma (arrows). **B**: The deposits stained with Masson’s trichrome showed brightly red accumulation of hyaline under the Bowman's membrane (arrows). The epithelium showed focal areas of dehiscence from Bowman's membrane.

A corneal button that was excised during a penetrating keratoplasty from the right eye of the proband demonstrated variably sized and irregularly shaped deposits within the corneal stroma. These deposits were situated mostly in the anterior and middle stroma and were adjacent to Bowman's membrane. The deposits stained positively with congo red in the anterior and middle stroma indicating be amyloid (Figure 4A) and staining with Masson’s trichrome showed bright red accumulation of hyaline under Bowman's membrane (Figure 4B). In addition, the epithelium showed focal areas of dehiscence from Bowman's membrane (Figure 4B).

## Discussion

In this study, we found an ACD family which exhibited a reduced penetrance of phenotype in northern China. As reported, ACD does not show complete dominance, and the homozygotes of R124H mutation display a more severe phenotype than the heterozygotes do [10,11]. In our family, the patients with the homozygous R124H mutation presented an early onset, severe corneal opacities and visual acuity had been impaired since childhood while the patients with the heterozygous mutation showed a late onset, mild corneal deposits and nearly no complaint about visual acuity. In addition, the expressivity among these heterozygotes was variable and it had no correlation with the age of patients. The father of the proband (I1)was mildly affected at 74 years of age. Moreover, we also found two individuals (II1,II6) who carried the same heterozygous R124H mutation presented no change to their corneal transparency and visual acuity at the age of 56 and 37, respectively. The 29-year-old daughter of II1 had initial symptoms at 22 and showed phenotypic characteristics corresponding to ACD. To avoid a missed diagnosis, the corneal status of all 7 unaffected (II1, II6, III6, III7, III9, III10, and III11) members who carried the heterozygous mutation were assessed by at least two investigators ignorant of the genotype. *TGFBI* exon 4 of these individuals was sequenced on both strands and repeated three times. The absence of disease in individual II1 most likely represents non-penetrance, rather than a missed diagnosis or a later onset of disease. At this point of view, our study observed a unique family of ACD which presented a reduced penetrance. This has not been previously reported in the study of ACD.

To date almost all cases studied with molecular genetic techniques for ACD have had the R124H mutation in *TGFBI* but the phenotype of affected individuals varies markedly in severity from family to family [10,14-17]. Watanabe et al. [18] observed two different clinical phenotypes of ACD in Japanese patients homozygous for the R124H mutation. In one variant a discrete grayish white opacity covered the anterior stroma and it was confluent in the central and paracentral cornea. In the other type, a reticular grayish white diffuse opacity was found in the anterior stroma of the cornea. Patients with the type II opacity traced their origin to Tottori prefecture in western Japan. They considered that even though the mutation in the gene responsible for the disease was the same, other genes and factors could modify the phenotype of the corneal opacity. In our family, patients with the homozygous *TGFBI* R124H mutation presented a similar phenotype to the type I from the Watanabe et al. [18] study. Moreover, a markedly variabile expressivity and apparent non-penetrance were observed in the individuals with heterozygous *TGFBI* R124H mutation in our pedigree. It indicated that there might be some modified genes or factors which could influence the phenotype of the causative gene.

It has been proven that modifier genes could play an important role in phenotypic variability in other monogenic disorders [19]. However, the genetic interactions between modifier genes and the mutated genes in corneal dystrophies is unclear. Watanabe et al. [18] explained the variable phenotype in patients with the homozygous *TGFBI* R124H mutation in their family for three possibilities. They considered that the *trans* acting genetic element, the *cis* acting genetic element, and environmental factors may affect the phenotype of the cornea with the homozygous *TGFBI* R124H mutation. In our case, the reduced penetrance of phenotype might be explained in the following ways. One possible reason may be the obtaining of a modified gene from the non-affected parent. This gene could ameliorate the effect of the mutated allele. Since such a protective gene could be absent in the offspring, it can not exhibit any protective effect in these individuals. Another explanation may be the loss of a modified gene from the affected parent, which could worsen the effect of the mutated allele. The final way is that the environment may affect the penetrance of mutated genes. But, our explanation about the genetic basis needs further determination. If those factors or genes could be identified, it may be possible to delay or inhibit the disease occurrence.

In summary, we reported a novel ACD family which exhibited a reduced penetrance of phenotype in northern China. The causative mutation was R124H in *TGFBI*. This outcome supports that although the R124H mutation is one of the genetic causes of the disease, different genetic and environmental factors may influence the expressivity and the penetrance. Uncovering the mechanism may lead to a way to inhibit the occurrence of the corneal dystrophy caused by R124H mutation in *TGFBI*, irrespective of the homozygous and heterozygous mutation.
